# Soluble CD83 alleviates experimental allergic rhinitis through modulating antigen-specific Th2 cell property

**DOI:** 10.7150/ijbs.38722

**Published:** 2020-01-01

**Authors:** Yong-Jin Wu, Yan-Nan Song, Xiao-Rui Geng, Fei Ma, Li-Hua Mo, Xiao-Wen Zhang, Da-Bo Liu, Zhi-Gang Liu, Ping-Chang Yang

**Affiliations:** 1 ENT Hospital of Shenzhen University School of Medicine, Longgang ENT Hospital, Shenzhen, China.; 2 Research Center of Allergy & Immunology, Shenzhen University School of Medicine, Shenzhen, China.; 3 Department of Otolaryngology, Head & Neck Surgery, First Affiliated Hospital of Guangzhou Medical University, Guangzhou, China.; 4 Department of Pediatric Otolaryngology, Shenzhen Hospital, Southern Medical University, Shenzhen, China.

**Keywords:** Allergy, nasal mucosa, CD83, CD4+ T cell, immune regulation

## Abstract

**Background and aims**: Dysfunction of the immune regulatory system plays a role in the pathogenesis of allergic rhinitis (AR). The underlying mechanism needs to be further investigated. Published data indicate that soluble CD83 (sCD83) has immune regulatory activities. This study aims to investigate the role of sCD83 in the alleviation of experimental AR.

**Methods**: Peripheral blood samples were obtained from AR patients. Serum levels of sCD83 were determined by enzyme-linked immunosorbent assay. A murine AR model was developed to test the effects of sCD83 on suppressing experimental AR.

**Results**: We found that serum levels of sCD83 in the AR group were lower than that in the healthy control group. A negative correlation was identified between the serum sCD83 levels and the frequency of T helper-2 (Th2) cells. The low serum sCD83 levels were also associated with the Bcl2L12 expression in antigen-specific Th2 cells. Exposure to sCD83 enhanced the responsiveness of antigen-specific Th2 cells to apoptosis inducers via suppressing the Bcl2L12 expression. Administration of sCD83 efficiently suppressed experimental AR.

**Conclusions**: sCD83 contributes to immune homeostasis by regulating CD4^+^ T cell activities. Administration of sCD83 may have translational potential for the treatment of AR or other allergic diseases.

## Introduction

Allergic rhinitis (AR) is one of the allergic diseases. AR is the adverse responses of the immune system in the nasal mucosa to airborne antigens (Ag), such as house dust mite (HDM). The AR clinical symptoms mainly include paroxysmal sneezing, profound nasal discharges and nasal congestion 1. Although AR symptoms are self-limited, it is reported prolonged AR attacks induce complications, such as chronic rhinosinusitis and nasal polyposis. Allergic asthma may be associated with AR 2. The AR prevalence is more than 10% in the world 3. The therapeutic efficacy is to be improved 4. In fact, AR attacks significantly affect human life quality and has been a health issue 1. Thus, it is necessary to further investigate the pathogenesis of AR, and invent novel and effective remedies for the treatment of AR.

The pathogenesis of AR is to be further investigated. It is the consensus that T helper-2 (Th2) polarization plays a crucial role in the development of AR. The Th2 polarization indicates a condition that the lesion tissues are over populated by Th2 cells that produce a large amount of Th2 cytokines, including interleukin (IL)-4, IL-5 and IL-13 5. One of the Th2 cytokine functions is to induce immunoglobulin-E (IgE) production by plasma cells. IgE binds the high affinity receptors on the surface of mast cells to make mast cells sensitized. Re-exposure to specific antigens activates the mast cells, induces mast cells to release allergic mediators, and triggers AR attacks 5. Yet, the mechanism of Th2 polarization is less understood. Remedies for adjusting Th2 polarization are limited currently.

CD83 is a member of the Ig superfamily. The CD83 gene is in Chromosome 6p23. The 5 exons of CD83 are: exon 1 is the leader sequence, exons 2 and 3 are the extracellular domain, exon 4 is the transmembrane domain and exon 5 is the intracellular domain 6. Mice also have the same gene organization as human 7. Most sCD83 is produced by B cells and dendritic cells (DC), and commonly used as a DC marker 8. CD83 can be released to the microenvironment to be the soluble CD83 (sCD83). sCD83 can be detected in healthy subjects; high sCD83 levels were found in some malignant tumors or autoimmune diseases 9, 10. It seems that sCD83 is involved in the immune regulation or is associated with immune disorders 11; whether sCD83 is associated with AR is to be investigated.

Our previous work indicates that the B cell lymphoma-2-like protein-12 (Bcl2L12), an apoptosis inhibitor, plays an important role in the development of Th2 polarization 12. Inhibition of Bcl2L12 efficiently suppresses Th2 response-related immune inflammation 12. Yet, the causative factors of Bcl2L12-over expression remain to be further investigated. On the other hand, sCD83 can regulate skewed immune responses 13. Whether the abnormal sCD83 levels is associated with the pathogenesis of AR is not clear. Therefore, we hypothesize that the abnormality of sCD83 levels may be associated with the pathogenesis of AR. In this study, AR patient serum sCD83 levels were assessed. The association between serum sCD83 levels and Th2 polarization in AR patients was analyzed. An AR murine model was developed to assess the role of sCD83 in the inhibition of experimental AR.

## Materials and Methods

### Patients

AR patients were recruited into this study at our clinic. The AR diagnosis was based on AR history, serum specific IgE ≥ 0.35 IU/ml, positive antigen skin test. The demographic data are presented in Table 1. The diagnosis and management were carried out by our physicians. Patients had any of the following conditions were excluded from this study: Autoimmune diseases; severe organ diseases; cancer; under treatment with corticosteroids for any reasons. The human experimental procedures were approved by the Human Ethic Committee at Shenzhen University. All the patients were recruited according to the approved ethic protocol. An informed written consent was obtained from each human subject.

### Enzyme-linked immunosorbent assay (ELISA)

Cytokine levels in the serum were determined by ELISA with commercial reagent kits, including specific IgE, mouse mast cell protease-1 (mMCP1), IL-4, IL-5 and IL-13, (BioMart; Beijing, China) following the manufacturer's instructions.

### Flow cytometry

Cells were collected from relevant experiments. In surface staining, cells were stained with fluorescence-labeled antibodies [CD3 (Alexa Fluor® 488), CD4 (Alexa Fluor® 546), IL-4 (Alexa Fluor® 594), IL-5 (Alexa Fluor® 647), IL-13 (Alexa Fluor® 680), CD154 (Alexa Fluor® 790), CD83 (FITC); Santa Cruz Biotech], [Bcl2L12 (APC); Abcam] (diluted at 1:100) or isotype IgG for 30 min on ice. In intracellular staining, cells were fixed with paraformaldehyde (1%; containing 0.1% Triton X-100) for 1 h and then stained with fluorescence-labeled antibodies of interest or isotype IgG for 30 min on ice. After washing with phosphate buffered saline (PBS) thoroughly, cells (10^6^ cells/sample) were analyzed with a flow cytometer (FACSCanto II). Data were analyzed with a software package flowjo (TreeStar Inc., Asland, OR) with the data obtained from isotype IgG staining as gating references.

### Real-time quantitative RT-PCR (RT-qPCR)

Cells were collected from relevant experiments. The total RNA was extracted from 10^7^ cells/sample with the TRIzol reagents and converted to cDNA with a reverse transcription kit following the manufacturer's instructions. The samples were amplified in a qPCR device with the SYBR Green Master Mix in the presence of relevant primers, including IRF1 (Forward: atgcttccacctctcaccaa. Reverse: tgtagctgctgtggtcatca), Bcl2L12 (human, forward: cttctatgctttggtggccc and reverse: tacagaacagctccaccagg; mouse, forward: ttccgagttctatgccctgg and reverse: ccagtttacgatgcagagcc). Results were processed with the 2^-∆∆Ct^ method and presented as relevant changes. (Reagents and materials for RT-qPCR were purchased from Invitrogen).

### Preparation of protein extracts

Cells (10^7^ cells/sample) were collected from relevant experiments and lysed with a lysis buffer (10 mM HEPES; 1.5 mM MgCl2; 10 mM KCl; 0.5 mM DTT; 1 mM EDTA; 0.05% NP40). The lysates were centrifuged at 13,000 g for 10 min. Supernatant was collected and used as the cytosolic extracts. The pellets were resuspended in a nuclear lysis buffer (5 mM HEPES; 1.5 mM MgCl_2_SO_4_; 4.6 M NaCl; 0.2 mM EDTA; 0.5 mM DTT; 26% glycerol), stayed for 30 min and centrifuged at 13,000 g for 10 min. Supernatant was collected and used as the nuclear extracts. Protein levels were determined by the BCA method. All the procedures were performed at 4 °C.

### Western blotting

Total proteins were extracted from cells collected from relevant experiments, separated by SDS-PAGE (4% V/V in the stacking gel and 10-12 % in the separating gel) at 50 µg/well and transferred onto a PVDF membrane. After blocking with skim milk (5%) for 30 min, the membrane was incubated with the primary antibodies [Bcl2L12 and IRF1 (Santa Cruz Biotech); diluted at 1:300] overnight at 4 °C, washed with TBST (Tris-buffered saline containing 0.1% Tween 20), incubated with peroxidase-conjugated secondary antibodies (diluted at 1:5000) for 2 h and washed with TBST 3 times. Immunoblots on the membrane were developed by the enhanced chemiluminescence and photographed in an imaging device (UVI; Cambridge, UK). (Reagents and materials for Western blotting were purchased from Invitrogen).

### Immunoprecipitation (IP)

Protein extracts were precleared by incubating with protein G agarose beads for 2 h. The beads were removed by centrifuging the samples at 5,000 g for 5 min. Supernatant was collected, a portion of the supernatant was collected and used as the input; the supernatant was incubated with antibodies [CD83 or CD154 (Santa Cruz Biotech); final concentration: 1:100] or isotype IgG overnight. Immunocomplexes in the samples were precipitated by incubating with protein G agarose beads for 2 h. The beads were collected by centrifugation. Proteins on the beads were eluted with an eluting buffer and analyzed by Western blotting. All the procedures were performed at 4 °C. (Reagents and materials for IP were purchased from Invitrogen).

### Chromatin immunoprecipitation (ChIP)

Cells (10^7^ cells/sample) were collected from relevant experiments, fixed with 1% paraformaldehyde for 15 min to cross-link the DNA with surrounding proteins. The samples were sonicated to shear the DNA into small pieces and precleared by incubating with protein G agarose beads for 2 h to remove pre-existing immune complexes. The beads were removed by centrifugation. Samples were incubated with antibodies of interest or isotype IgG overnight. Immune complexes were precipitated by incubating with protein G agarose beads for 2 h. The beads were collected by centrifugation. Immune complexes on the beads were eluted with an eluting buffer. DNA was recovered from the samples with a DNA extracting kit following the manufacturer's instructions and analyzed by qPCR in the presence of Bcl2L12 promoter primers (Forward: tcctgcacttaccccaatgt and reverse: gaggaaaagaggaggcgtct). The results were presented as fold change against the input. All the procedures were performed at 4 °C. (Reagents and materials for ChIP were purchased from Invitrogen).

### Isolation of mononuclear cells from the airway tissues

Mouse nasal mucosa and lungs were collected, cut into small pieces (2 × 2 × 2 mm) and incubated with collagenase IV (1 mg/ml; Sigma Aldrich) at 37 °C for 2 h with mild agitation. Single cells were filtered through a cell strainer (70 µm first, then 40 µm). Mononuclear cells were isolated from the single cells by Percoll gradient density centrifugation.

### Cell culture

Cells (10^6^ cells/ml) were cultured in RPMI1640 medium supplemented with fetal calf serum (10%), antibiotics (penicillin, 100 U/ml; streptomycin, 0.1 mg/ml) and L-glutamine (2 mM). Cell viability was greater than 99% as assessed with by Trypan blue exclusion assay. Cell culture materials were purchased from Sigma Aldrich.

### Preparation of house dust mite (HDM) protein

HDM (*Dermatophagoides farina*) was prepared in our own laboratory as reported previously 14.

### Isolation of Ag-specific Th2 cells (sTh2 cells)

Blood samples were collected from AR patients (sensitized to HDM). PBMCs were isolated from the samples by gradient density centrifugation. Preexisting CD154^+^ (an activation marker of T cells 15) cells were selected out by flow cytometry cell sorting (FCCS). The cells were then cultured in the presence of HDM (5 µg/ml; prepared in our own laboratory) overnight. The cells were stained with fluorescence-labeled antibodies of CD3, CD4 and CD154 (Santa Cruz Biotech). The CD3^+^ CD4^+^ CD154^+^ T cells were isolated by FCSS. As assessed by flow cytometry, more than 90% CD4^+^ CD154^+^ T cells were also IL-4^+^. These T cells were regarded as the sTh2 cells.

### Clustered regularly interspaced short palindromic repeats (CRISPR) or RNA interference (RNAi)

sTh2 cells were prepared as described above and treated with CD154 CRISPR reagents, or IRF1 RNAi reagents (Santa Cruz Biotech), or control reagents to depleting CD154 or IRF1 expression, following the manufacturer's instructions. The effects of CRISPR and RNAi were assessed 48 h later by Western blotting.

### Assessment of apoptotic cells

Cells were collected from relevant experiment and stained with propidium iodide (PI) and an annexin v reagent kit (Sigma Aldrich) following the manufacturer's instruction. The cells were then analyzed by flow cytometry. The annexin v^+^ cells or PI^+^ annexin v^+^ cells were regarded as apoptotic cells.

### Mice

Male C57BL/6 mice (6-8-week old) were purchased from Guangdong Experimental Animal Center (Guangzhou, China). CD154-deficient mice were purchased from Jackson Laboratory (Bar Harbor, ME). Mice were maintained in a specific pathogen-free facility with accessing water and food freely. The animal experimental procedures were approved by the Animal Ethic Committee at Shenzhen University.

### AR mouse model development

Mice were subcutaneously injected with HDM (0.1 mg/mouse, mixed in 0.1 ml alum; prepared in our laboratory) on day 0, day 3 and day 7, respectively. Nasal instillation with HDM (5 mg/ml in saline) at 50 µl/nostril was carried out daily from day 15 to day 24.

### Treating AR mice with sCD83

From day 25, AR mice were treated with nasal instillation containing sCD83 (0.1 mg/ml; purchased from Sangon Biotech; Shanghai, China. This dosage was tested in the preliminary studies; data not shown) or saline (control) at 50 µl/nostril daily for 7 days.

### Assessment of AR response in AR mice

Following our established procedures 16, AR responses were assessed in each mouse, including AR symptoms (sneezing and nasal scratching), serum levels of specific IgE and mouse mast cell protease-1 (mMCP1), Th2 cytokine amounts in nasal tissue protein extracts and nasal epithelial barrier permeability to FITC-dextran (Sigma Aldrich). (Levels of specific IgE, mMCP1, Th2 cytokines were assessed by ELISA following the manufacturer's instruction; the reagents were purchased from R&D Systems).

### Statistics

Data are presented as mean ± SEM. Student *t* test was performed to determine the difference between two groups. ANOVA followed by Dunnett's test or Bonferroni test was performed for multiple comparisons. The Pearson correlation assay was performed to determine the correlation between parameters. P<0.05 was set as a significant criterion.

## Results

### Serum sCD83 levels are lower in AR patients and negatively correlated with serum specific IgE

**Figure 1:**
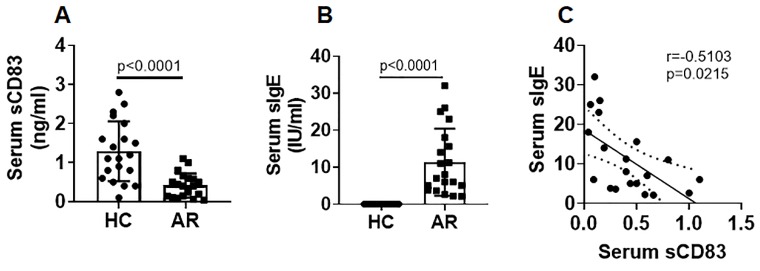
** AR serum sCD83 is negatively correlated with serum sIgE**. Blood samples were collected from 20 healthy control (HC) subjects and 20 allergic rhinitis (AR) patients. The serum was prepared from the samples and analyzed by ELISA. **(A)** serum sCD83 levels. **(B)** serum sIgE levels (by ImmunoCap). **(C)** a negative correlation between serum sCD83 and serum sIgE. Data of bars are presented as mean ± SEM. Each dot in bars present data obtained from individual subjects (in duplicate). Statistics of A and B: Mann Whitney test. C: Pearson correlation assay.

We collected blood samples from AR patients and healthy control (HC) subjects. The serum was isolated from the samples and analyzed by ELISA. The results showed that sCD83 levels were significantly lower in the AR group (median: 0.4; 0.04~1.1 ng/ml) than that in the HC group (median: 1.15; 0.4~2.8 ng/ml) (Fig. 1A). Furthermore, ELISA results showed that specific IgE (sIgE) was detected in the serum of AR patients (Fig. 1B). We found that serum sCD83 levels were negatively correlated with serum sIgE (Fig. 1C). The results suggest that sCD83 may be associated with the pathogenesis of AR.

### Serum sCD83 levels are associated with Th2 polarization

**Figure 2:**
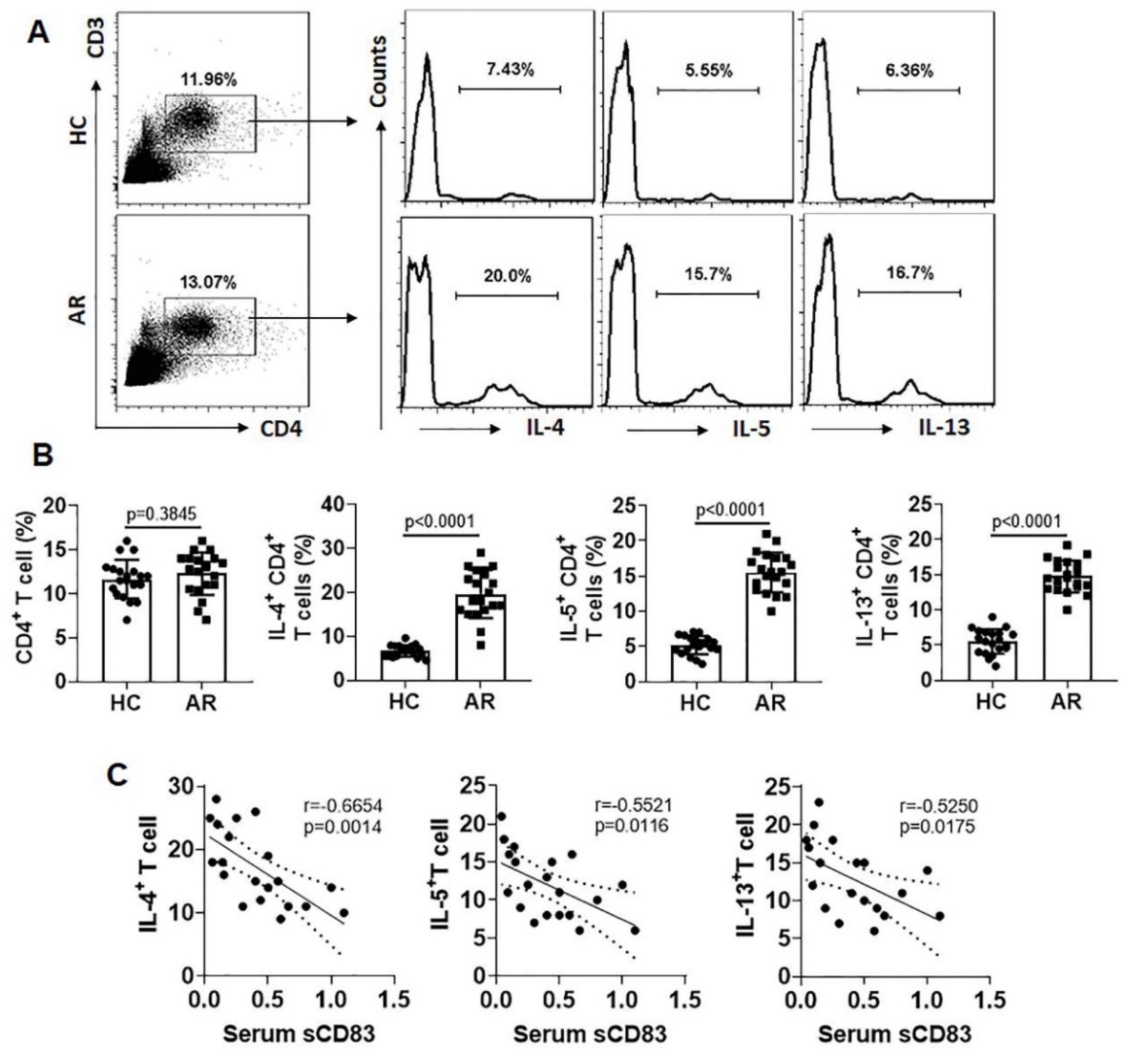
** Serum sCD83 levels are negatively correlated with Th2 polarization in AR patients.** Blood samples were collected from 20 HC subjects and 20 AR patients. Peripheral blood mononuclear cells (PBMC) were isolated and analyzed by flow cytometry. **(A)** Gated dot plots show frequency of CD3^+^ CD4^+^ T cells. Gated histograms show frequency of Th2 cells. **(B)** summarized data of panel A. **(C)** correlation between serum sCD83 (data are presented in Fig.1) and Th2 cells. Data of bars are presented as mean ± SEM. Each dot in bars present data obtained from one patient. Statistics: *t* test (B) or Pearson correlation assay (C).

Since Th2 polarization plays a critical role in the AR pathogenesis 5, we next assessed a possible link between the serum sCD83 levels and the phenomenon of Th2 polarization in AR patients. The Th2 polarization was detected in the AR group manifesting higher frequency of Th2 cells in the peripheral blood system (Fig. 2A-B). A negative correlation was detected between serum sCD83 and Th2 cells (Fig. 2C). The results suggest that the low serum sCD83 levels may be associated with the pathogenesis of Th2 polarization in AR.

### Serum levels of sCD83 negatively correlate with Bcl2L12 expression in antigen-specific Th2 cells (sTh2 cells)

**Figure 3:**
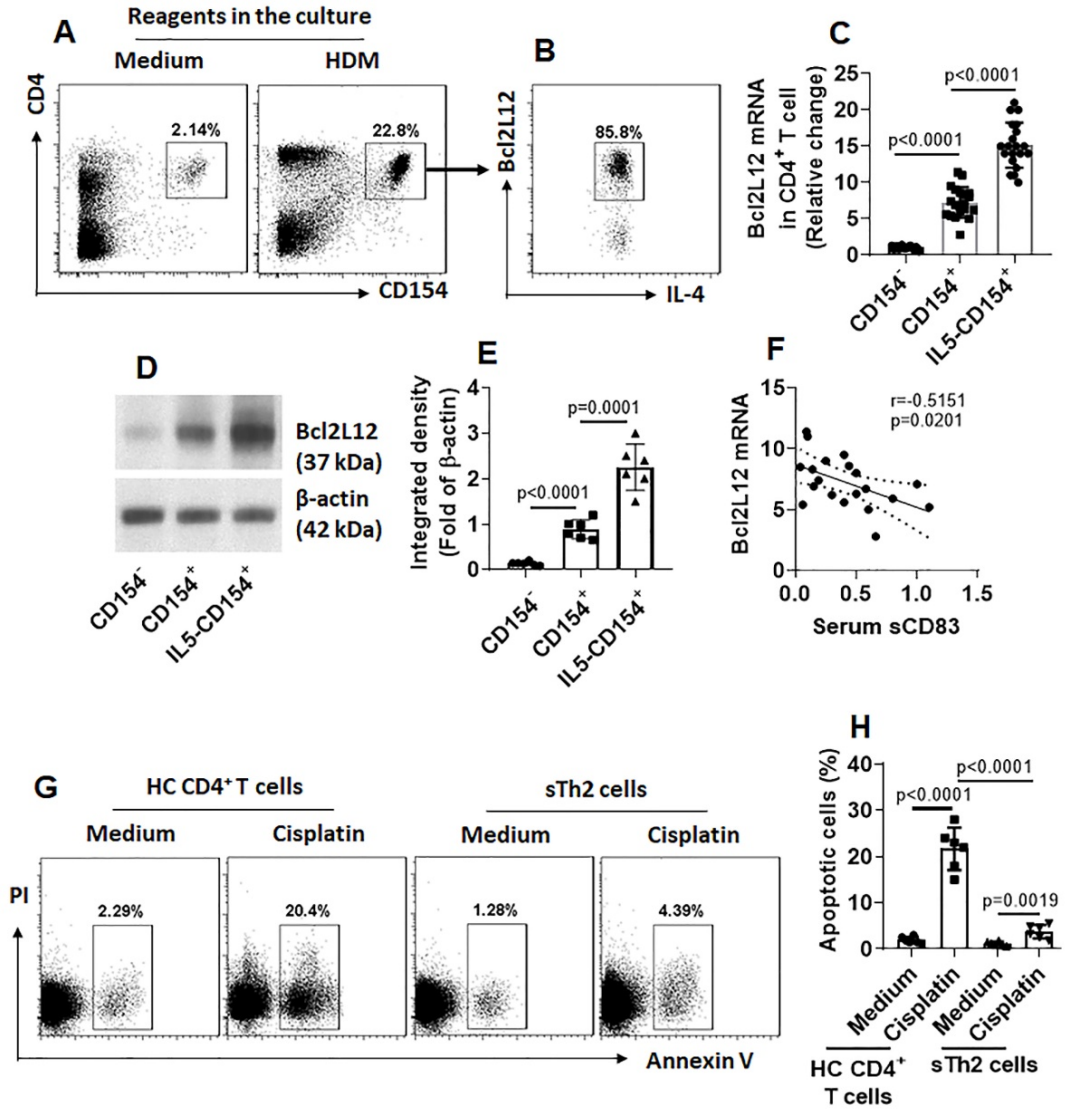
** A negative correlation between serum sCD83 levels and Bcl2L12 expression in Ag-specific Th2 cells.** CD4^+^ T cells and DCs were isolated from AR patient PBMCs and cultured with specific Ag (HDM, 5 µg/ml; this dosage was tested in the preliminary studies; data not shown) for 16 h. **(A-B)** dot plots in upper right quadrant show CD154^+^ CD4^+^ T cells (A); these cells also express IL-4 and Bcl2L12 (B). **(C-E)** CD154^+^ CD4^+^ T cells and CD154^+^ CD4^+^ T cells were isolated by FCCS and analyzed by RT-qPCR and Western blotting. **(C)** Bcl2L12 mRNA levels in CD4^+^ CD154^+^ T cells. IL5: CD4^+^ CD154^+^ T cells were isolated by FCCS and cultured in the presence of IL-5 (20 ng/ml) for 48 h. **(D)** Bcl2L12 protein levels in CD4^+^ CD154^+^ T cells. **(E)** Immunoblot image analysis data of panel D. **(F)** A negative correlation between serum sCD83 levels (data are presented in Fig. 1) and Bcl2L12 mRNA in CD154^+^ CD4^+^ T cells (cultured with HDM for 16 h). **(G)** Gated dot plots indicate apoptotic cells. **(H)** Summarized apoptotic cells of panel G. Data of bars are presented as mean ± SEM. Each dot in bars presents data obtained from one sample. Statistics: ANOVA (C, E and H: p<0.0001) followed by Bonferroni test. Pearson correlation assay: Panel F.

Our previous studies indicate that Bcl2L12 plays a critical role in the pathogenesis of Th2 polarization 17. We wondered if the low serum sCD83 levels were also associated with the over expression of Bcl2L12 in sTh2 cells. To this end, we firstly assessed the expression of Bcl2L12 in sTh2 cells. In line with our previous work 17, we also detected highly expression of Bcl2L12 in sTh2 cells (Fig. 3A-E). Exposure of sTh2 cells to IL-5 16 in the culture further increased Bcl2L12 expression in the cells (Fig. 3C-E). A negative correlation between serum sCD83 levels and Bcl2L12 expression was detected in sTh2 cells (Fig. 3F). In line with our previous findings 17, sTh2 cells also showed apoptosis resistance (Fig. 3G-H). The results suggest that the low levels of sCD83 are associated with the over expression of Bcl2L12 in sTh2 cells.

### sCD83 binds CD154 on sTh2 cells to restrict Bcl2L12 expression

**Figure 4:**
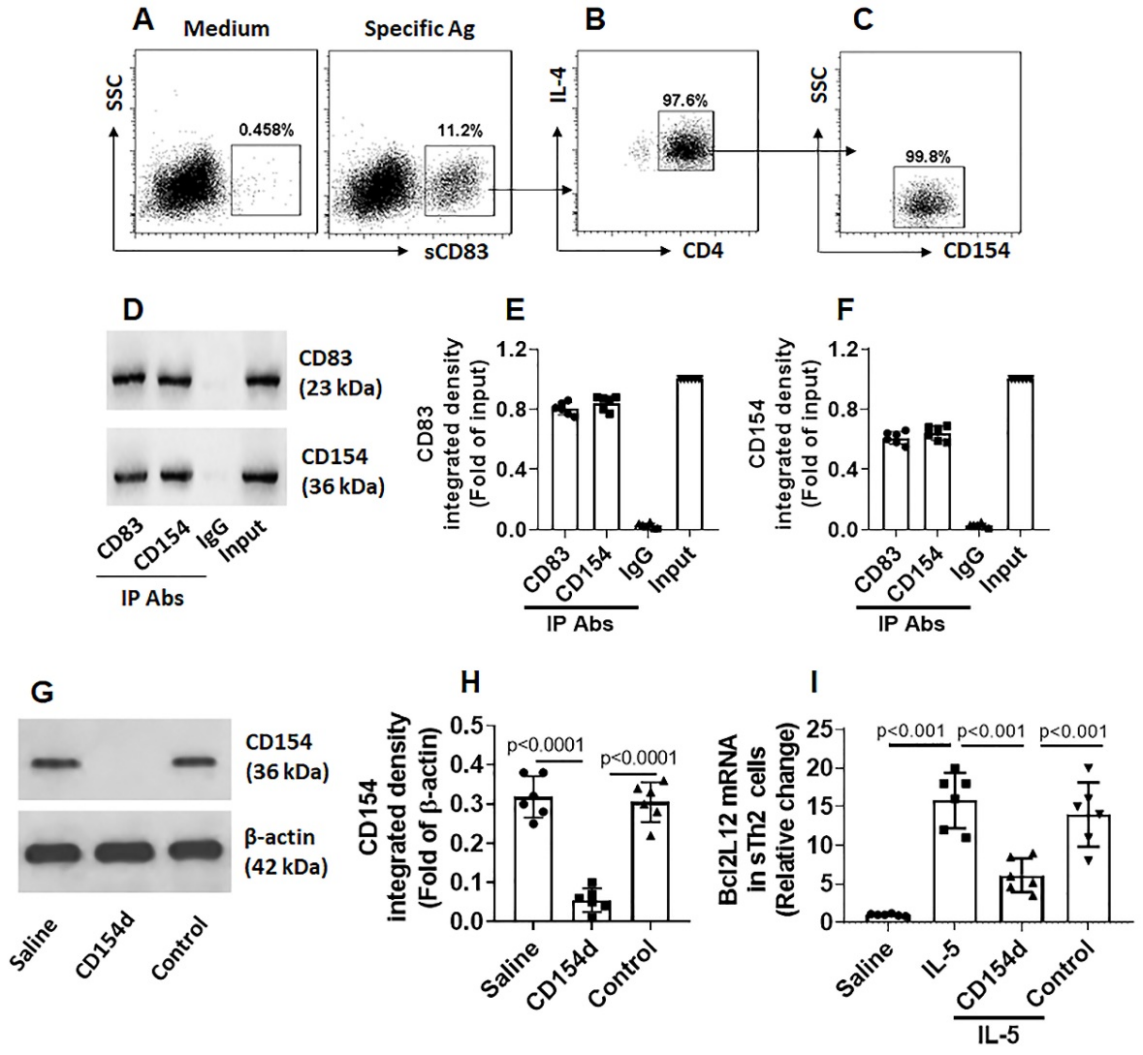
** sCD83 binds CD154 on the surface of sTh2 cells**. **(A)** PBMCs were isolated from blood samples obtained from AR patients (sensitized to HDM) and cultured in HDM (5 µg/ml) overnight, and then exposed to sCD83 (labeled with FITC) in the culture for 6 h. The cells were stained with anti-CD4 and IL-4 antibodies, and analyzed by flow cytometry. Gated dot plots show the sCD83-labeled cells (indicate that the sCD83 bound to a portion of PBMCs); these cells were also CD4^+^ IL-4^+^ **(B)** and CD154^+^ **(C). (D)** CD83^+^ CD154^+^ cells were isolated by FCCS and analyzed by co-IP. Immunoblots show a complex of CD83/CD154. **(E-F)** Bars show immunoblot image analysis data of panel D. **(G)** Immunoblots show results of CD154-depletion in sTh2 cells. **(H)** bars show immunoblot image analysis data of panel G. **(I)** Bars show Bcl2L12 mRNA levels in sTh2 cells. Data of bars are presented as mean ± SEM. Each dot in bars presents data obtained from each experiment. CD154d: CD154-deficient sTh2 cells (prepared by CD154 CRSPR). Control: sTh2 cells were treated with control CRISPR reagents. IL-5: IL-5 in the culture (20 ng/ml). Statistics of H-I: ANOVA (p<0.0001) followed by Bonferroni test. Data represent 6 independent experiments.

Fig. 3 shows that sCD83 suppresses the Bcl2L12 expression in sTh2 cells. We next investigated the underlying mechanism. By exposing freshly isolated PBMCs to sCD83 in the culture, less than 1% CD4^+^ T cells were bound by sCD83. However, after exposing to specific Ags in the culture, the frequency of sCD83-bound cell was markedly increased (Fig. 4A); these were also CD4^+^ IL-4^+^ (Fig. 4B), as well as CD154^+^ (Fig. 4C), indicating that these cells are sTh2 cells. The results suggest that sCD83 ligands exist on the surface of sTh2 cells that only appear upon activation. As shown by Fig. 3, CD154 appear on the surface of sTh2 cells after exposure to specific Ags; other investigators also found this phenomenon 15. Thus, we hypothesize that CD154 is the ligand of sCD83. To this end, after exposing to specific Ags in the culture, CD4^+^ CD154^+^ T cells were isolated by FCCS and exposed to sCD83 in the culture. Six hours later, the cells were collected and analyzed by immunoprecipitation. A complex of CD83/CD154 was detected (Fig. 4D-F). The results indicate that sCD83 can bind to CD154 and such binding results in suppression of Bcl2L12 expression in sTh2 cells. To test this, CD4^+^ CD154^+^ sTh2 cells were isolated by FCCS. A portion of sTh2 cells were depleted the CD154 expression by CRISPR (Fig. 4G-H). The cells were then stimulated by IL-5 16 in the culture in the presence or absence of sCD83. Exposure to IL-5 markedly increased the Bcl2L12 expression in sTh2 cells; the Bcl2L12 expression was suppressed by the presence of sCD83, that was abolished by the depletion of CD154 (Fig. 4I). The results indicate that CD154 is a ligand of sCD83. By binding CD154, sCD83 inhibits Bcl2L12 expression in sTh2 cells.

### sCD83 up regulates interferon regulatory factor 1 (IRF1) expression to suppress Bcl2L12 expression in sTh2 cells

**Figure 5:**
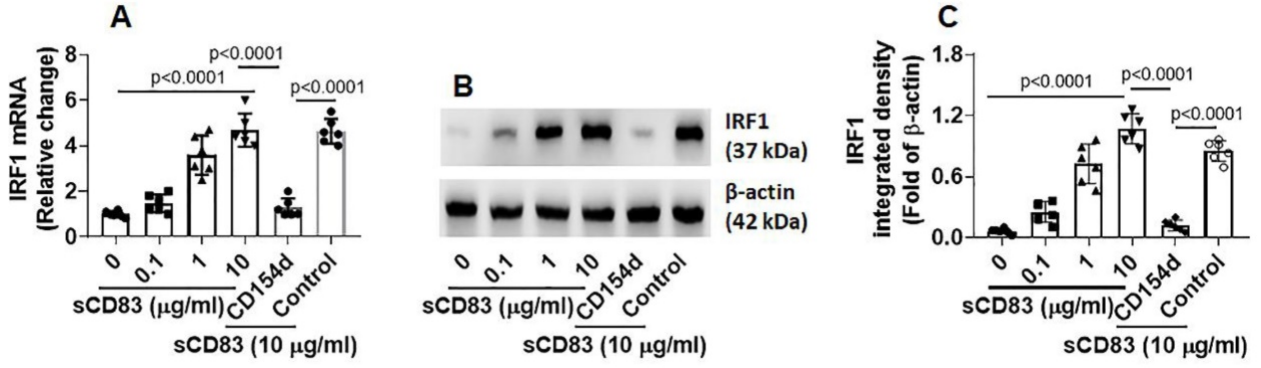
** sCD83 increases IRF1 expression in sTh2 cells**. sTh2 cells were prepared from blood samples collected from 6 AR patients. The sTh2 cells were treated with the procedures denoted on the x axis of panel A. **(A)** Bars indicate IRF1 mRNA levels in sTh2 cells. CD154d: CD154-deficient sTh2 cells (prepared by CRISPR). Control: sTh2 cells were treated with control CRISPR reagents. Data of bars are presented as mean ± SEM. Each dot in bar present data obtained from one independent experiment. Statistics: ANOVA or *t* test. **(B)** Proteins extracted from the 6 sTh2 cell samples were pooled and analyzed by Western blotting (repeated 3 times); immunoblots show IRF1 proteins in sTh2 cells (representing 3 independent experiments). **(C)** Immunoblot image analysis data of panel B.

Published data indicate that IRF1 is the transcription factor of interferon (IFN)-γ and plays a critical role in triggering Th1 response 18, and Bcl2L12 contributes to Th2 polarization development 16, 17. Thus, we hypothesize that sCD83 may up regulate IRF1 expression to suppress Bcl2L12 expression in sTh2 cells. To test this, sTh2 cells were prepared and cultured in the presence of sCD83 for 48 h. Indeed, the expression of IRF1 was increased by sCD83 in a concentration-dependent manner that could be blocked by depleting the CD154 expression (Fig. 5). The results indicate that sCD83 ligates CD154 to induce IRF1 expression in sTh2 cells.

**Figure 6:**
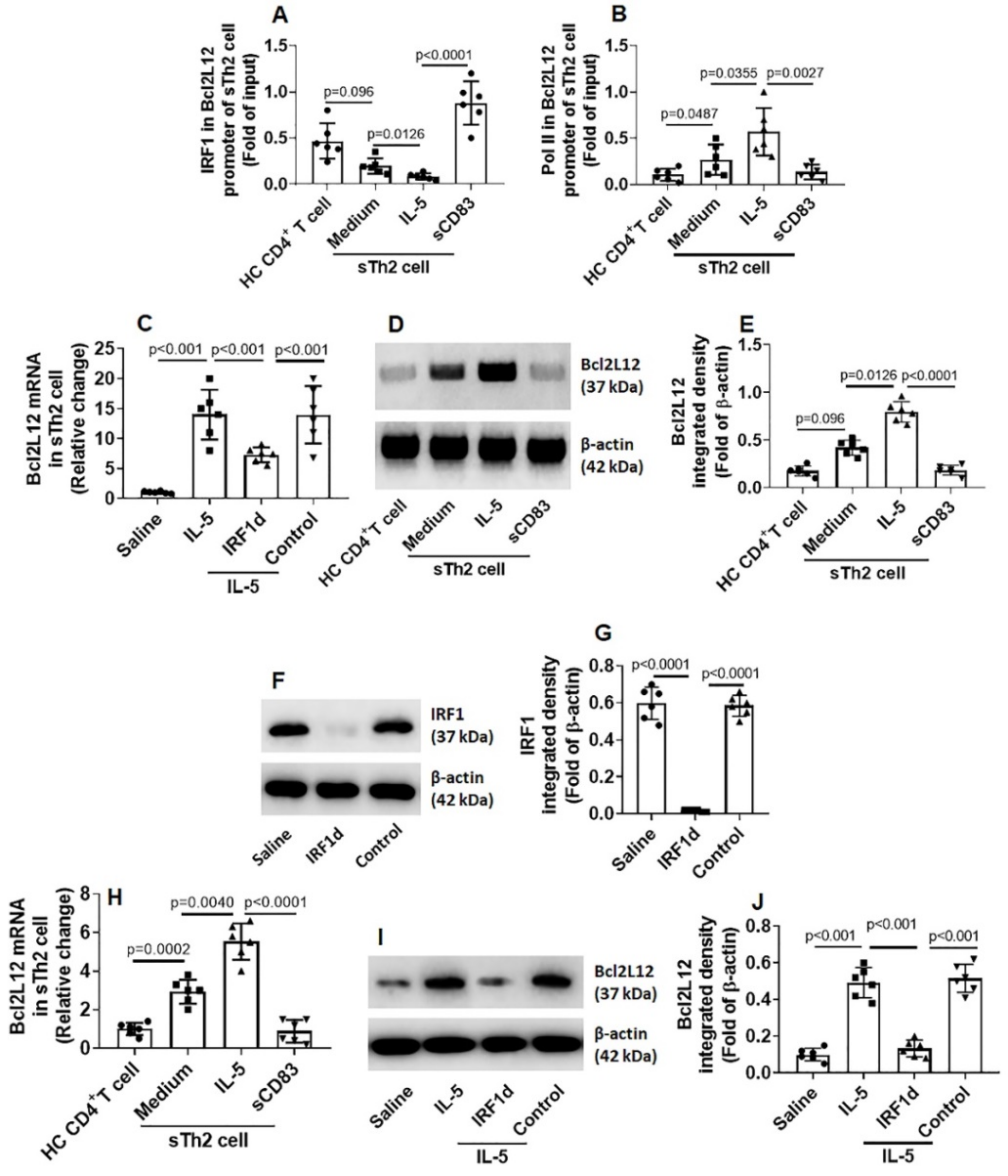
** IRF1 represses Bcl2L12 expression in sTh2 cells. (A-D)** sTh2 cells were isolated from blood samples from AR patients; naïve CD4^+^ T cells were collected from HC subjects. The cells were treated with procedures denoted on the x axis of panel A. Bars indicate levels of IRF1 (A) and Pol II (B) in the Bcl2L12 promoter (by ChIP). Bars indicate Bcl2L12 mRNA levels (C). Immunoblots indicate Bcl2L12 protein (D). **(E)** Immunoblot image analysis data of panel D. **(F)** Results of IRF1 CRISPR in sTh2 cells. IRF1d: IRF1-deficiency. **(G)** Immunoblot image analysis data of panel F. **(H-J)** sTh2 cells were treated with the procedures denoted on the x axis of panel H. Bars show Bcl2L12 mRNA levels (H). Immunoblots show Bcl2L12 protein levels (I). Immunoblot image analysis data of panel I (J). Statistics: ANOVA + Bonferroni test.

After exposing to sCD83, an increase in the amounts of IRF1 was detected in the promoter of Bcl2L12 (Fig. 6A). Such an effect was in parallel to the amounts of RNA polymerase II (Pol II; an indicator of gene transcription activity 19) in the Bcl2L12 promoter (Fig. 6B) and Bcl2L12 expression in sTh2 cells (Fig. 6C-E). The results suggest that IRF1 is a repressor of Bcl2L12. To verify the role of IRF1 in mediating the effects of sCD83 on suppressing Bcl2L12, we depleted IRF1 expression in sTh2 cells by CRISPR (Fig. 6F-G). The IRF1-deficient sTh2 cells were exposed to sCD83. Indeed, depletion of IRF1 abolished the sCD83-induced Bcl2L12 suppression in sTh2 cells (Fig. 6H-J). The data suggest that sCD83 ignites a signal pathway in sTh2 cells. The signal pathway components include CD154, IRF1 and Bcl2L12. Deletion of any of the components abolished the inhibitory effects of sCD83 on the Bcl2L12 expression in sTh2 cells.

### Administration of sCD83 alleviates experimental AR

**Figure 7:**
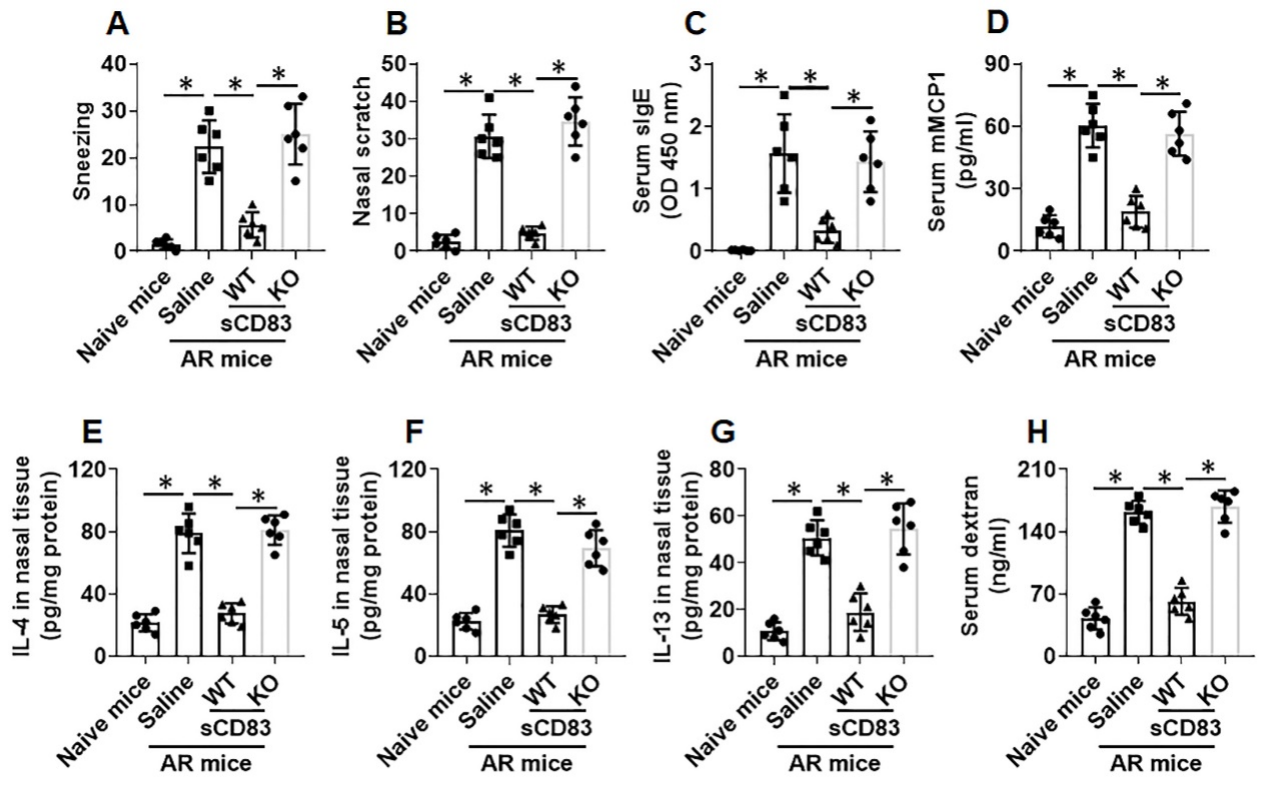
** Administration of sCD83 alleviates experimental AR.** AR mice were treated with either sCD83 or saline via nasal instillation daily for one week. The mice were challenged with specific antigens one day after the last treatment. AR responses were recorded and assessed. **(A-B)** AR nasal symptoms, including sneezing and nasal scratching (nasal itch) within 1 h after the challenge. **(C-D)** Serum levels of specific IgE (sIgE) and mMCP1 (released from mast cells). **(E-G)** Th2 cytokine amounts in nasal tissue protein extracts (Assessed by ELISA). **(H)** Serum levels of FITC-dextran (an indicator of nasal epithelial barrier permeability). WT: Wild type mice (C57BL/6 mice). KO: CD154-deficient mice (C57BL/6 background). Data of bars are presented as mean ± SEM. Each dot in bars present data obtained from one mouse. *p<0.001 (ANOVA followed by Bonferroni test).

Next, an AR murine model was developed. We found that administration of sCD83 efficiently suppressed the AR symptoms and AR-related immune pathological responses in the mice, which were abolished by depleting the CD154 expression in mice (Fig. 7).

**Figure 8:**
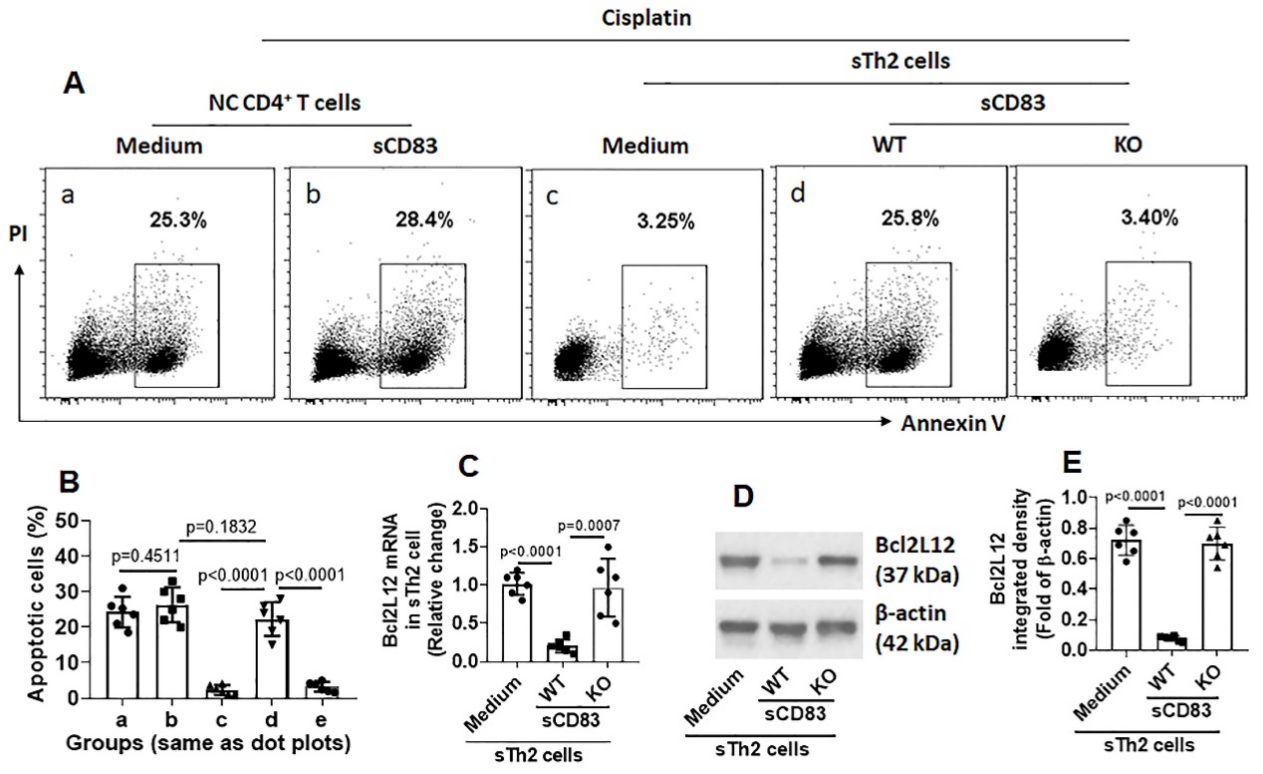
** sCD83 inhibits Bcl2L12 expression to increase the responsiveness of sTh2 cells to apoptosis inducers.** Naïve control (NC) mice and AR mice were treated with sCD83 or saline daily for one week. Mononuclear cells were isolated from the airway tissues (nasal mucosa and lungs) and exposed to HDM (5 µg/ml) and cisplatin (25 µM) overnight. The cells were then analyzed by flow cytometry. **(A)** Gated dot plots show apoptotic cell frequency. **(B)** Summarized apoptotic cells in panel A. **(C-E)** Expression of Bcl2L12 in sTh2 cells. WT: Wild type mice (C57BL/6 mice). KO: CD154-deficient mice (C57BL/6 background). Data of bars are presented as mean ± SEM. Each dot in bars present data obtained from one mouse. Each group consists of 6 mice. Statistics: ANOVA (p<0.0001) followed by Bonferroni test.

**Figure 9:**
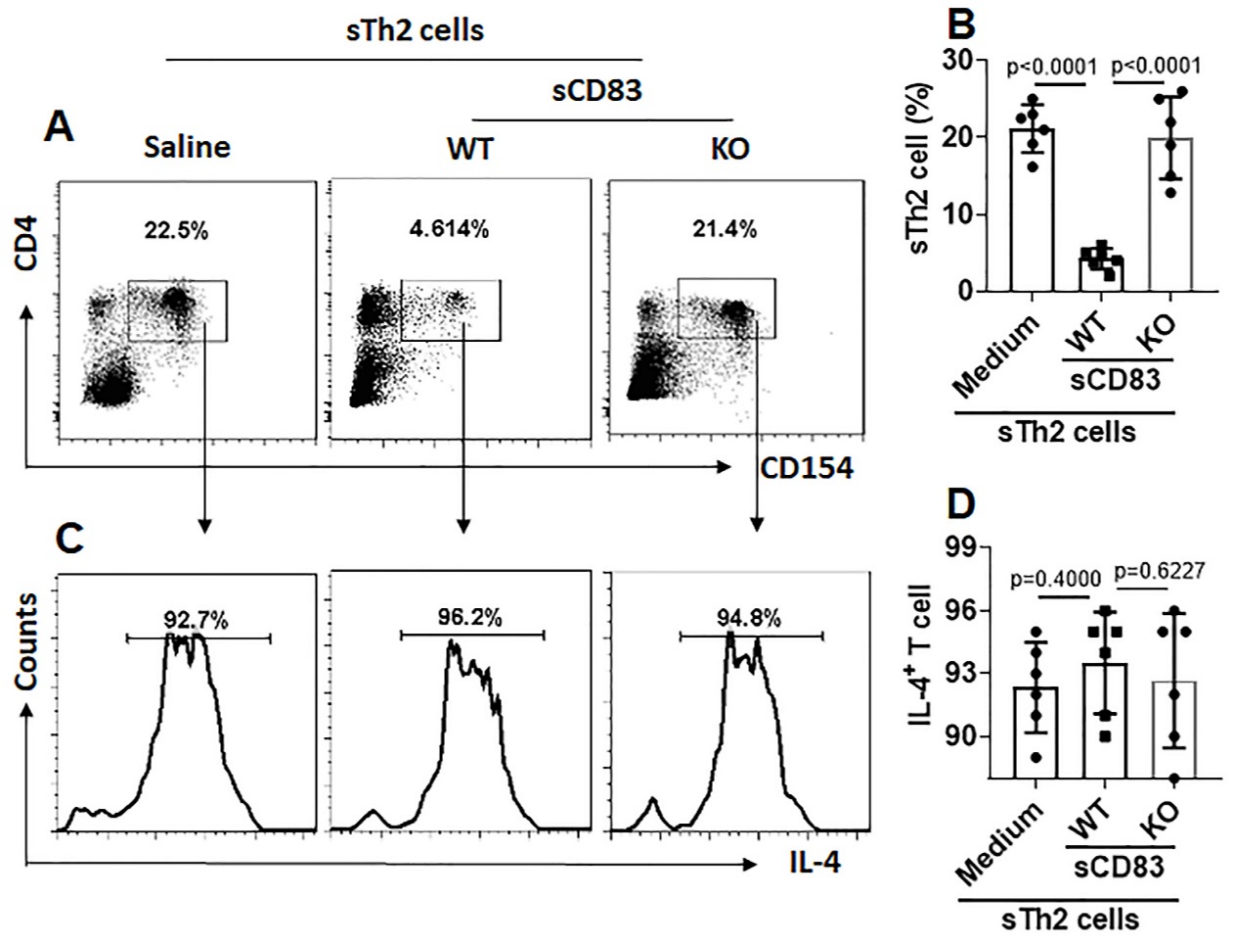
** sCD83 reduces sTh2 cells in the airway tissues**. Mononuclear cells were isolated from the airway tissues of AR mice and cultured in the presence of HDM (5 µg/ml) overnight. The cells were analyzed by flow cytometry. **(A)** Gated dot plots show the HDM-activated CD4^+^ T cells. **(B)** Summarized data of panel A. **(C)** Gated histograms show IL-4^+^ cells in the HDM-activated CD4^+^ T cells of panel A. **(D)** Summarized data of panel C. WT: Wild type mice (C57BL/6 mice). KO: CD154-deficient mice (C57BL/6 background). Data of B and D are presented as mean ± SEM. Each dot in bars present data obtained from one mouse. Each group consists of 6 mice. Statistics: ANOVA (p<0.0001) followed by Bonferroni test.

We noticed that, sTh2 cells showed significantly lower responsiveness to apoptosis inducer that was markedly increased after treating with sCD83 (Fig. 8A-B). The expression of Bcl2L12 in sTh2 cells was markedly reduced by treating with sCD83 (Fig. 8C-E). The frequency of sTh2 cells in the airway tissues was significantly reduced (Fig. 9). Depletion of CD154 abolished the effects of sCD83. The results demonstrate that administration of sCD83 can suppress experimental AR by reducing sTh2 cell population in the airway tissues.

## Discussion

With the increase in CD154 as a marker of Ag-specific activation, we were able to isolate sTh2 cells to be characterized. We found that sCD83 could bind CD154 to sTh2 cells to increase the expression of IRF1, the latter suppressed the expression of Bcl2L12 in sTh2 cells, and thus, suppressed Th2 polarization and inhibited established AR in a murine model.

We found that AR patients had lower serum sCD83 levels. This is negatively correlated with the status of Th2 polarization. The results implicate that the reduction of sCD83 may contribute to the pathogenesis of airway allergy. This phenomenon was also reported by others that serum sCD83 levels were lower in patients with multiple sclerosis 20. Administration of sCD83 restricted autoimmune antigen-induced arthritis by suppressing the levels of proinflammatory cytokines such as IL-17A, IFN-γ, IL-6, and TNF-α in the joint tissues 21. In a murine colitis model, Eckhardt et al found that sCD83 decreased inflammatory cytokine expression in mesenteric lymph nodes and colon, inhibition of the infiltration of macrophages and granulocytes into colonic tissues, and induced expression of indolamine 2,3-dioxygenase to fulfill its protective effects 22. Our data add novel information to this research spot by showing that AR patients also have lower serum levels of sCD83 that is associated with the AR pathogenesis.

It is the consensus that Th2 polarization plays a critical role in the pathogenesis of AR. Current understanding about the pathogenesis of Th2 polarization is that this disorder is caused by multiple factors. For example, nematode infection is a common cause of Th2 polarization 23. Microbial products are another cause to induce Th2 polarization, such as cholera toxin is used in the development of food allergy animal model 24. The present data show that the low serum sCD83 levels are negatively correlated with the Th2 response in AR patients, suggesting that the low serum sCD83 levels are associated with the pathogenesis of Th2 polarization.

Previous reports show that CD4^+^ T cells from patients with allergic diseases express high levels of Bcl2L12 16, 17, the present data also show that sTh2 cells express high Bcl2L12 levels. Bcl2L12 is an apoptosis inhibitor. Cells with high Bcl2L12 levels are resistant to apoptosis inducers 12. The data show that sTh2 cells of subjects with allergic diseases show apoptosis resistance as well as high levels of Bcl2L12. Inhibition of Bcl2L12 can increase the sensitiveness of CD4^+^ T cells to apoptosis inducers 12. The present data reveal another aspect of this phenomenon. The high expression of Bcl2L12 in sTh2 cells negatively correlates with lower serum sCD83 levels in AR patients, indicating that the decrease in sCD83 may be an important factor associating with the high expression of Bcl2L12 in sTh2 cells.

Although the ligands of sCD83 have not been determined, many reports indicate that sCD83 can bind immune cells to fulfill the immune regulatory activities 25, indicating potential sCD83 ligands on the surface of immune cells. Our data reveal a ligand of sCD83, the CD154. CD154 is an activation marker of immune cells 15. It is also the ligand of CD40; thus, it is also called CD40 ligand. In line with previous reports, we found CD154 was specifically up regulated in CD4^+^ T cells upon being activated by exposure to specific Ags. These cells are sTh2 cells since these cells also expressed IL-4. Therefore, after exposure to specific Ags, the CD4 and CD154 can be specific markers to isolate sTh2 cells.

From sCD83 to the Bcl2L12 suppression, a signal transduction pathway was identified in the present study. We found that sCD83 activated CD154 and IRF1 in Ag-specific Th2 cells sequentially. Since IRF1 was found in the promoter of Bcl2L12, the amounts of IRF1 in the Bcl2L12 promoter were reversely associated with the Bcl2L12 mRNA levels in sTh2 cells, we believe that IRF1 is the executive molecule to repress the Bcl2L12 expression. By blocking any of the components, the suppressive effects of sCD83 on Bcl2L12 expression were abolished. The results verify that sCD83 suppresses Bcl2L12 expression in sTh2 cells through this signal transduction pathway.

The therapeutic remedies for AR include Ag-specific immunotherapy (SIT), anti-histamines, corticosteroids, etc. Although SIT is expected to fundamentally treat AR, the therapeutic efficacy needs to be improved. Other remedies, such as anti-histamines and corticosteroids, only temporary stop AR attacks. The present data suggest that sCD83 has the translational potential to be used as a therapeutic agent for AR through suppressing the expression of Bcl2L12 in sTh2 cells to restore the responsiveness to apoptosis inducers, and thus, suppressing the Th2 polarization status.

In summary, the present data show that AR patients have low serum sCD83 levels. Administration of sCD83 inhibits AR response and AR-related immune pathological changes through suppressing Bcl2L12 and up regulating IRF1 expression in sTh2 cells.

## Figures and Tables

**Table 1:** Demographic data

	AR patients	Healthy subjects
Number	20 (male 10, female 10)	20 (male 10, female 10)
Age (years)	34.5 ± 5.3	33.2 ± 3.8
Height	161.5 ± 6.5	162.2 ± 4.5
Body weight	58.5 ± 8.7	57.8 ± 6.3
AR history (years)	2.2 ± 1.1	None
HDM SPT positive	20	None
Serum total IgE (IU/ml)	322 ± 125	0.22 ± 0.1
AR/asthma	4 (20%)	0
AR/eczema	3 (15%)	0
AR/food allergy	1 (5%)	0
Current smoker	0	0

AR: Allergic rhinitis; HDM: House dust mite. Serum IgE levels were determined by ImmunoCap.
